# Frequentist and Bayesian approaches for food allergen risk assessment: risk outcome and uncertainty comparisons

**DOI:** 10.1038/s41598-019-54844-1

**Published:** 2019-12-03

**Authors:** Sophie Birot, Amélie Crépet, Benjamin C. Remington, Charlotte B. Madsen, Astrid G. Kruizinga, Joseph L. Baumert, Per B. Brockhoff

**Affiliations:** 1 DTU Compute, Richard Petersens Plads, DK-2800 Kgs, Lyngby, Denmark; 2 ANSES, French Agency for Food, Environmental and Occupational Health Safety, 14 rue Pierre et Marie Curie, 94701 Maisons-Alfort, France; 3 The Netherlands Organization for Applied Scientific Research (TNO), Zeist, The Netherlands; 4 National Food Institute, Technical University of Denmark, Lyngby, Denmark; 5 Food Allergy Research and Resource Program, Department of Food Science & Technology, University of Nebraska, 143 Food Industry Complex, Lincoln, Nebraska 68583-0919 United States

**Keywords:** Risk factors, Public health

## Abstract

Peer-reviewed probabilistic methods already predict the probability of an allergic reaction resulting from an accidental exposure to food allergens, however, the methods calculate it in different ways. The available methods utilize the same three major input parameters in the risk model: the risk is estimated from the amount of food consumed, the concentration of allergen in the contaminated product and the distribution of thresholds among allergic persons. However, consensus is lacking about the optimal method to estimate the risk of allergic reaction and the associated uncertainty. This study aims to compare estimation of the risk of allergic reaction and associated uncertainty using different methods and suggest improvements. Four cases were developed based on the previous publications and the risk estimations were compared. The risk estimation was found to agree within 0.5% with the different simulation cases. Finally, an uncertainty analysis method is also presented in order to evaluate the uncertainty propagation from the input parameters to the risk.

## Introduction

Food allergy has been a growing public health concern over the last decade with an estimated prevalence of around 3–5% in adults and up to 8% in children^1^. People with food allergies need to avoid consuming food products containing the offending allergen^2^ to prevent allergic reactions. To support this, ingredient labelling should provide essential information to allergic individuals on which allergens are present in the food product as an ingredient. However, unexpected allergic reactions can still occur due to the unintended presence of allergen in food products, as a result of e.g. manufacturing processes. In order to alert allergic consumers and avoid potentially dangerous allergic reactions, food manufacturers use precautionary allergen labelling (PAL) when cross contamination is suspected or facilities are shared^3^.

The increasing use of PAL and a lack of understanding of its application has led people with food allergies to disregard the statements^4^, putting themselves at risk of a reaction. Good practice in the effective application of PAL by manufacturers dictates that it must rest on a risk assessment focussing on the health risk to the consumer, preferably quantitative^5^. An international workshop under the auspices of the Europrevall project agreed that probabilistic modelling was the most promising approach for assessing the risk from allergens at the population level^6^. Thus, the health consequences of unintended presence of allergens in food products i.e the probability of provoking a reaction in the susceptible population can be expressed quantitatively and this information can be used to evaluate possible risk mitigation measures, for example PAL or different forms of expressing it.

Several research groups have developed probabilistic risk assessment approaches for food allergens^7–9^. The approaches described have the same three input model variables: the consumption distribution (i.e. how much of the suspected contaminated product is consumed), the concentration distribution (i.e. how much allergen is in the contaminated product) and the threshold distribution (i.e. the range of minimum doses of allergen eliciting reactions in the relevant food-allergic population). One of the modelling methods is based on second order Monte Carlo simulations^9^ and the others are based on the combination of Bayesian inferences and second order Monte-Carlo simulations^7,8^. Based on these previous approaches, four different cases were developed to estimate the risk of an unexpected allergic reaction. This paper aims to compare the risk estimates under these four different cases and to understand the mechanism of uncertainty propagation from the input parameters to the risk when using different simulations methods. Specifically, a case was built based on log normal distributions for all the input variables. For this case, it is also possible to calculate mathematically the uncertainty around the risk estimate and to compare with results from simulations.

## Materials and Methods

All four cases presented in the method have the same input distribution, namely the consumption distribution (X), the concentration distribution (Y) and the threshold distribution (Z). The probability of allergic reaction is then calculated from the three distributions (p(Z < XY)) at the population level and in a different way for each case. The differences between the four cases are summarized in Table 1.

**Table 1:** Summary of the different case presented to assess the uncertainty propagation from the inputs to the risk of allergic reaction.

Input	Distribution	Case A	Case B		Case C	Case D
		Frequentist – No uncertainty	Triple Log-Normal – Simulated	Triple Log-Normal – Calculated	Frequentist derived from case D	Bayesian
Consumption (X)	Parameter	No distribution	${{\rm{\mu }}}_{{\rm{X}}}\sim {\mathscr{N}}({{\rm{\mu }}}_{{\rm{Xl}}},\frac{{{\rm{\sigma }}}_{{\rm{X}}}}{\sqrt{{{\rm{n}}}_{{\rm{X}}}}})$ ${S}_{X}^{2}\sim \frac{{\sigma }_{X}^{2}}{{n}_{X}-1}{\chi }^{2}({n}_{X}-1)$		${{\rm{\mu }}}_{{\rm{X}}}\sim {\mathscr{N}}({{\rm{\mu }}}_{{\rm{Xl}}},\frac{{{\rm{\sigma }}}_{{\rm{X}}}}{\sqrt{{{\rm{n}}}_{{\rm{X}}}}})$ ${S}_{X}^{2}\sim \frac{{\sigma }_{X}^{2}}{{n}_{X}-1}{\chi }^{2}({n}_{X}-1)$	Bootstrap
	Input	Log-Normal ${\rm{X}}\sim {\mathcal L} {\mathscr{N}}({{\rm{\mu }}}_{{\rm{X}}},{{\rm{\sigma }}}_{{\rm{X}}})$	Log-Normal ${\rm{X}}\sim {\mathcal L} {\mathscr{N}}({{\rm{\mu }}}_{{\rm{X}}},{{\rm{\sigma }}}_{{\rm{X}}})$	Calculated	Log-Normal ${\rm{X}}\sim {\mathcal L} {\mathscr{N}}({{\rm{\mu }}}_{{\rm{X}}},{{\rm{\sigma }}}_{{\rm{X}}})$	Empirical
Contamination (Y)	Parameter	No distribution	${{\rm{\mu }}}_{{\rm{Y}}}\sim {\mathscr{N}}({{\rm{\mu }}}_{{\rm{Yl}}},\frac{{{\rm{\sigma }}}_{{\rm{Y}}}}{\sqrt{{{\rm{n}}}_{{\rm{Y}}}}})$ ${S}_{Y}^{2}\sim \frac{{\sigma }_{Y}^{2}}{{n}_{Y}-1}{\chi }^{2}({n}_{Y}-1)$		${{\rm{\lambda }}}_{{\rm{Y}}}\sim {\mathscr{N}}({{\rm{\mu }}}_{{\rm{Y}}},\frac{{{\rm{\sigma }}}_{{\rm{Y}}}}{\sqrt{{{\rm{n}}}_{{\rm{Y}}}}})$	${{\rm{\lambda }}}_{{\rm{Y}}}\sim {\rm{Gamma}}\,({\rm{\alpha }}+{{\rm{n}}}_{{\rm{Y}}},{\rm{\beta }}+\mathop{\sum }\limits_{{\rm{j}}=1}^{{{\rm{n}}}_{{\rm{y}}}}\,{{\rm{Y}}}_{{\rm{j}}})$ With vague prior parameters α = β = 10^−3^
	Input	Log-Normal ${\rm{Y}}\sim {\mathcal L} {\mathscr{N}}({{\rm{\mu }}}_{{\rm{Y}}},{{\rm{\sigma }}}_{{\rm{Y}}})$	Log-Normal ${\rm{Y}}\sim {\mathcal L} {\mathscr{N}}({{\rm{\mu }}}_{{\rm{Y}}},{{\rm{\sigma }}}_{{\rm{Y}}})$	Calculated	Exponential Y ~ Exp (λ_Y_)	Exponential Y ~ Exp (λ_Y_)
Threshold (Z)	Parameter	No distribution	${{\rm{\mu }}}_{{\rm{Z}}}\sim {\mathscr{N}}({{\rm{\mu }}}_{{\rm{Zl}}},\frac{{{\rm{\sigma }}}_{{\rm{Z}}}}{\sqrt{{{\rm{n}}}_{{\rm{Z}}}}})$ ${S}_{Z}^{2}\sim \frac{{\sigma }_{Z}^{2}}{{n}_{Z}-1}{\chi }^{2}({n}_{Z}-1)$		${{\rm{a}}}_{{\rm{Z}}}\sim {\mathscr{N}}(\widehat{{{\rm{\mu }}}_{{{\rm{a}}}_{{\rm{Z}}}}},\widehat{{{\rm{\sigma }}}_{{{\rm{a}}}_{{\rm{Z}}}}})$ ${{\rm{b}}}_{{\rm{Z}}}\sim {\mathscr{N}}(\widehat{{{\rm{\mu }}}_{{{\rm{b}}}_{{\rm{Z}}}}},\widehat{{{\rm{\sigma }}}_{{{\rm{b}}}_{{\rm{Z}}}}})$	Bayesian inferences with vague priors: a_Z_ ~ Gamma (10^−3^, 10^−3^) b_Z_ ~ Gamma (10^−3^, 10^−3^)
	Input	Log-Normal ${\rm{Z}}\sim {\mathcal L} {\mathscr{N}}({{\rm{\mu }}}_{{\rm{Z}}},{{\rm{\sigma }}}_{{\rm{Z}}})$	Log-Normal ${\rm{Z}}\sim {\mathcal L} {\mathscr{N}}({{\rm{\mu }}}_{{\rm{Z}}},{{\rm{\sigma }}}_{{\rm{Z}}})$	Calculated	Weibull Z ~ Weibull (a_Z_, b_Z_)	Weibull Z ~ Weibull (a_Z_, b_Z_)

### Mathematical formulation of the existing approaches

#### Mathematical formulation of the model developed by Spanjersberg *et al*.^9^

The probabilistic risk assessment method described in Spanjersberg *et al*.^9^ aims to take into consideration the variability and the uncertainty from input variables and can express the risk for the allergic user population (i.e. all individuals in the risk assessment simulation are allergic to the allergen of interest and consume the contaminated product). The allergic-user population risk can be expressed with a mathematical formulation and the three input distributions are stated in the article as:

$${{\rm{X}}}_{{\rm{ik}}}\sim \,\mathrm{Log} \mbox{-} \mathrm{Normal}\,({{\rm{\mu }}}_{{\rm{X}}},{{\rm{\sigma }}}_{{\rm{X}}})\,({\rm{Consumption}}\,{\rm{distribution}})$$

$${{\rm{Y}}}_{{\rm{ik}}}\sim \,\mathrm{Log} \mbox{-} \mathrm{Normal}\,({{\rm{\mu }}}_{{\rm{Y}}},{{\rm{\sigma }}}_{{\rm{y}}})\,({\rm{Concentration}}\,{\rm{distribution}})$$

$${{\rm{Z}}}_{{\rm{ik}}}\sim {\rm{empirical}}\,{\rm{distribution}}\,{\rm{of}}\,{\rm{threshold}}$$

where *i*(=*1*, …, *n*) is the iteration and *k*(=*1*, …, *K*) the replication.

The simulations are then repeated for *n* iterations and *K* replications from which the uncertainty and variability associated with the risk estimate are calculated. For each (i, k), X_ik_, Y_ik_ and Z_ik_ are simulated from the distributions, if X_ik_Y_ik_ > Z_ik_, then U_ik_ = 1, otherwise U_ik_ = 0. Thus, the risk is calculated for each run *k*:

$${R}_{k}=\frac{1}{n}\mathop{\sum }\limits_{i=1}^{n}\,{U}_{ik}$$

And the standard deviation is calculated for the K risks:

$$sd=\sqrt{\mathop{\sum }\limits_{k=1}^{K}\,\frac{{({R}_{k}-\bar{R})}^{2}}{K-1}}$$

Originally the uncertainty and variability associated with the risk estimate were taken into account by adding uncertainty on the scale parameters (σ) of the Log-Normal consumption and concentration distributions and by adding variability among persons for the threshold distribution. However, the parameters do not vary from replication to replication (k). Increasing the number of simulations, i.e. increase in the number of iterations and/or replications, will lead to a more accurate estimate of the risk. However, the standard deviation calculated by this method expresses both the uncertainty of the numerical procedure and the uncertainty introduced by the input variables. Furthermore, the model was improved to take into account the uncertainty associated with the input parameters as described in Remington *et al*.^8,10^ but this is not further discussed in the current study.

#### Mathematical formulation of the model developed by Rimbaud *et al*.^7^

The probabilistic risk assessment described in Rimbaud *et al*.^7^ uses a combination of second order Monte-Carlo simulations and Bayesian inferences to estimate the risk of allergic reaction. The consumption (X), the contamination (Y) and the threshold (Z) distributions can also be used to express mathematically the risk in the allergic user population. In Bayesian analysis, the parameters characterizing the input distributions are themselves represented by distributions. Thus, the actual input distributions and the distribution of their prior parameters are defined below:

$${{\rm{X}}}_{{\rm{ik}}}\sim {\rm{empirical}}\,{\rm{distribution}}\,{\rm{with}}\,{\rm{sampling}}\,{\rm{with}}\,{\rm{replacement}}\,{\rm{with}}\,\mathrm{survey}\mbox{'}s\,{\rm{weights}}$$

$${{\rm{Y}}}_{{\rm{ik}}}\sim {\rm{Exponential}}\,({{\rm{\lambda }}}_{{\rm{k}}})\,{\rm{with}}\,{\rm{prior}}\,{\rm{distribution}}:{\rm{\lambda }}\sim {\rm{Gamma}}\,({10}^{-3},{10}^{-3})$$

$${{\rm{Z}}}_{{\rm{ik}}}\sim {\rm{Weibull}}\,({{\rm{a}}}_{{\rm{k}}},{{\rm{b}}}_{{\rm{k}}})\,{\rm{with}}\,{\rm{prior}}\,{\rm{distribution}}:({\rm{a}},{\rm{b}})\sim {\rm{Gamma}}\,({10}^{-3},{10}^{-3})$$

The distributions of the posterior parameters are evaluated either by direct calculation when prior distributions are conjugate or using Markov chain Monte-Carlo simulations. This results in parameter distributions from which K set of parameters are sampled in order to integrate the uncertainty in the risk estimation and n iterations are made for each set of parameters. The posterior parameter distributions are obtained using specific datasets that will be presented in the results section. Then, the risk is estimated at the individual level from the threshold dose distribution:

$${{\rm{Risk}}}_{{\rm{ik}}}=\mathrm{Dose} \mbox{-} \mathrm{Response}\,{(\mathrm{Exposure}}_{{\rm{ik}}})\,{\rm{with}}\,{{\rm{Exposure}}}_{{\rm{ik}}}={{\rm{X}}}_{{\rm{ik}}}\times {{\rm{Y}}}_{{\rm{ik}}}$$

The risk is defined as the probability that the allergic consumer will react to the amount of allergen ingested. Thus, for each set of parameters, distributions of risks are obtained from the n individual results to describe the uncertainty and variability in risks. Case D is inspired by the Rimbaud *et al*.^7^ approach.

#### Methods comparison

The two approaches developed by Spanjersberg *et al*.^9^ and Rimbaud *et al*.^7^ have the same input variables namely consumption, concentration and threshold distribution. But, as described in sections 2.1.1 and 2.1.2, the two models use the input distribution in different ways to estimate the risk of allergic reaction. After mathematical review, it was highlighted that the method in Spanjersberg *et al*.^9^ is not able to separate the simulation uncertainty and the uncertainty integrated by the input parameters. However, the method described in Rimbaud *et al*.^7^ makes explicit the uncertainty propagation from the input variables to the risk calculation. More detailed investigation on uncertainty propagation with these two approaches are explained in section 2.4.

Besides the uncertainty around the risk estimates, the two methods also differ from the way the threshold curve is used. In Spanjersberg *et al*.^9^, for each individual a threshold is simulated from the density function and then directly compared to the amount of allergen ingested. The risk is calculated by counting the number of reactions among the simulated consumers. In contrast, in Rimbaud *et al*.^7^, the dose response curve (cumulative density function) is used to predict the chance of an allergic reaction for each consumer considering their exposure. The distribution of resulting risk estimates is used to estimate the overall risk of allergic reaction and its uncertainty. When the same threshold data are used, the difference in use of this distribution should not impact the risk estimation by the two methods and this is illustrated succinctly in Appendix 1 by estimating the risk using both the density and the cumulative density functions. Finally, this point will not be further addressed as it is not the main focus of the paper.

### Mathematical formulation of the alternative frequentist approach and description of the cases under study

From a frequentist perspective, the risk is a deterministic function of the parameters from the three distributions, exemplified later in the article by the triple-log-normal case. The risk estimated from the three data sources is hence the same function with parameters estimated by sampling from each data source (the parameter estimates are then used in the risk function). Thus, the sample uncertainty of this risk estimate can be seen as a straightforward error propagation challenge for the risk function. Namely, the uncertainty which propagates from each sampling parameter estimate is evaluated through the non-linear risk function to provide an uncertainty evaluation of the risk estimate. In this paper, simulations were used to assess the uncertainty propagation with normal approximation for mean estimates and chi-square distributions for variance parameter estimates^11^.

#### Mathematical formulation of the alternative frequentist approach

Inspired from Spanjersberg *et al*.^9^, Monte-Carlo simulations that include parameter sampling can be used. The parameter distributions are defined from well-known distributions. The parameters of the consumption, concentration and threshold distribution are estimated from the data. It is assumed that their parameters are defined with the observations from a random sample of size *l* following the normal distribution *N*(*μ*, *σ*^2^):

- The sample mean of the distributions parameter is sampled from a normal distribution with mean μ_l_ and standard deviation $\frac{\sigma }{\sqrt{l}}$ (uncertainty on the mean)^11^. So, the mean is sampled from: ${\rm{\mu }}\sim {\mathscr{N}}({{\rm{\mu }}}_{l},\frac{\sigma }{\sqrt{l}})$.
- When a lognormal distribution is used, the sample variance S^2^ can be directly simulated from its specific distribution: for a sample of size *l* and with σ^2^ variance is follows a chi-square distribution: $\frac{(l-1){S}^{2}}{{\sigma }^{2}}\sim {\chi }_{l-1}^{2}$ ^11^. So, the variance is sampled from: ${S}^{2} \sim \frac{{\sigma }^{2}}{l\,-1}{\chi }^{2}(l-1)$ and is used as the scale parameter of the lognormal distribution.
- In some cases, a model is fitted to the input data in order to estimate the distribution of the parameters and the associated uncertainty. The parameters are then sampled from normal distribution with the mean of the estimated parameter and standard deviation of the parameters estimated with the regression. So, the parameter a is sampled from: ${\rm{a}}\sim {\mathscr{N}}(\widehat{{{\rm{\mu }}}_{{\rm{a}}}},\widehat{{{\rm{\sigma }}}_{{{\rm{a}}}_{a}}})$.

K different sets of parameters (number of replications) are sampled from these distributions as defined in Table 1 to assess the uncertainty around the risk.

#### Case A (Spansbjerg) and Case B (calculated and stimulated): Triple log-normal case

In case A (Spansbjerg), the risk calculation is based on from the probabilistic risk assessment described in Spanjersberg *et al*.^9^. Three log-normal distributions are used for the three input parameters (consumption – X, contamination – Y, threshold – Z) and no uncertainty around the three inputs was integrated in the risk calculation.

In Case B (triple log-normal), three log-normal distributions are again used to fit the three input parameters. Each lognormal distribution is described with its location *μ* and scale *σ* parameter. Additionally, the uncertainty is integrated by sampling the distribution parameters from normal and chi-squared distributions as defined in 2.2.1. The risk of allergic reaction can then be expressed mathematically and is then a function of 6 parameters:

$${p}_{u}=f({\mu }_{x},{\sigma }_{x},{\mu }_{y},{\sigma }_{y},{\mu }_{z},{\sigma }_{z})$$

In fact, in this case it becomes:

$${p}_{u}=1-\Phi (\frac{{\mu }_{z}-{\mu }_{x}-{\mu }_{y}}{\sqrt{{({\sigma }_{x})}^{2}+{({\sigma }_{y})}^{2}+{({\sigma }_{z})}^{2}}})$$

where φ is the probability density function of the standard normal distribution.

This risk expression will also be used to evaluate the uncertainty propagation both mathematically and with simulations. Thus, the comparison done in the triple log normal (Case B) will allow us to investigate the additivity of the different sources of uncertainty.

#### Case C: Frequentist derived from Rimbaud *et al*.^7^

Distributions were selected in order to compare the risk estimation and the uncertainty propagation estimated in Case C (frequentist inferences and simulations) with Case D (Rimbaud: simulations and Bayesian inferences). Thus, the actual input distributions and the distribution of their parameters for Case C are defined below:

$${{\rm{X}}}_{{\rm{ik}}}\sim {\rm{Lognormal}}\,{({\rm{\mu }}}_{{\rm{k}}},{{\rm{\sigma }}}_{{\rm{k}}})\,{\rm{with}}\,{\rm{sampling}}\,{\rm{distribution}}\,{\rm{defined}}\,\mathrm{2.2.1}$$

$${{\rm{Y}}}_{{\rm{ik}}}\sim {\rm{Exponential}}\,{({\rm{\lambda }}}_{{\rm{k}}})\,{\rm{with}}\,{\rm{sampling}}\,{\rm{distribution}}:{{\rm{\lambda }}}_{{\rm{k}}}\sim {\mathscr{N}}\,({{\rm{\mu }}}_{{\rm{Y}}},\frac{{{\rm{\sigma }}}_{{\rm{Y}}}}{\sqrt{{{\rm{l}}}_{{\rm{Y}}}}})$$

$${\rm{Zik}}\sim {\rm{Weibull}}\,{(a}_{{\rm{k}}},{{\rm{b}}}_{{\rm{k}}})\,{\rm{with}}\,{\rm{sampling}}\,{\rm{distribution}}\,{\rm{defined}}\,\mathrm{2.2.1}$$

K set of parameters are then sampled from the distribution of each parameters in order to integrate the uncertainty in the risk estimation and n iterations are made for each set of parameters.

#### Case D: Bayesian based on Rimbaud *et al*.^7^

Case D is designed in the same way as Rimbaud *et al*.^7^ and was previously described in 2.1.2.

### Comparison of different cases to estimate the risk of an unexpected allergic reaction

#### General risk calculation

The four cases are formulated to estimate the risk of an unexpected allergic reaction. The cases are formulated in the same way, so the similarities and differences between the various ways of simulating from the distribution can be explicitly clarified and the consequences for the risk assessment can be formally assessed. Especially, the differences in the way the uncertainty propagates with all the simulation methods will be assessed. The consumption *X* follows a distribution noted *F*_*X*_, the concentration *Y* follows a distribution called *F*_*Y*_ and the threshold *Z* follows a distribution called *F*_*Z*_. The allergy outcome *U* is defined as follow:

$$\{\begin{array}{l}{\rm{U}}=1,\,{\rm{if}}\,{\rm{Z}} < {\rm{XY}},\,{\rm{and}}\\ {\rm{U}}=0\,{\rm{o}}{\rm{t}}{\rm{h}}{\rm{e}}{\rm{r}}{\rm{w}}{\rm{i}}{\rm{s}}{\rm{e}}\end{array}$$

**Figure 1:**
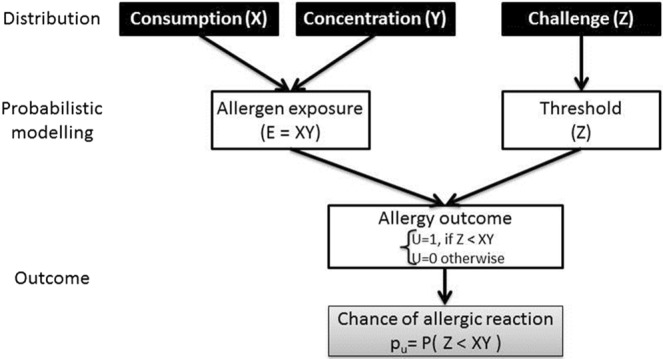
Risk estimation – general principle.

U follows a Bernoulli distribution with probability p_u_, where p_u_ = P(Z < XY). Formally it is assumed that the three random variables X, Y and Z are independent. The probability p_u_ is mathematically a function of the three distributions: *p*_*u*_ = *f*(*F*_*x*_, *F*_*y*_, *F*_*z*_). The general principle of the risk modelling is presented in Fig. 1.

If these three distributions are given by parameters, then this is a function of the following:

$${p}_{u}=f({\theta }_{x},{\theta }_{y},{\theta }_{z})$$

#### Sampling distributions for the different cases

For each case, the distribution chosen for each input and the distribution of their parameters are presented in Table 1. The same simulation framework as described in 2.3.1 is used so the risk estimation can be directly compared.

### Uncertainty propagation

In order to assess how the uncertainty propagates from the consumption, contamination and threshold distributions separately to the risk, an adapted version of the uncertainty analysis described previously^12^ was used. This method is compliant with EFSA recommendations to evaluate sources of uncertainty individually^13^.

#### General principle – mathematical formulation

Propagation of the sampling uncertainty of all the distributions through the non-linear ${\hat{p}}_{u}$ computation was investigated. This can actually be investigated for each distribution separately or for all of them jointly. Thus, it makes it possible to identify which input variable adds the most uncertainty to the risk. Formally, it amounts to considering the ${\hat{p}}_{u}$ as a random variable as a function of the data:

$${\hat{P}}_{u}^{X}=f({\hat{\Theta }}_{X},{\hat{\theta }}_{y},{\hat{\theta }}_{z})$$

where ${\hat{\Theta }}_{X}$ is a random sampling statistic which investigated the uncertainty induced by consumption data sampling, and the other two are held at the observed estimated parameters. Or, to investigate the uncertainty induced by the concentration data sampling:

$${\hat{P}}_{u}^{Y}=f({\hat{\theta }}_{x},{\hat{\Theta }}_{Y},{\hat{\theta }}_{z})$$

Or, to investigate the uncertainty induced by the threshold data sampling:

$${\hat{P}}_{u}^{Z}=f({\hat{\theta }}_{x},{\hat{\theta }}_{y},{\hat{\Theta }}_{Z})$$

Or all of them, which is how the risk of allergic reaction is usually calculated in probabilisitic modelling. The uncertainty induced by the three distributions is investigated:

$${\hat{P}}_{u}=f({\hat{\Theta }}_{X},{\hat{\Theta }}_{Y},{\hat{\Theta }}_{Z})$$

#### Mathematical formulation of uncertainty propagation for Case B (Triple-log-normal)

The uncertainty around the risk can indeed be calculated with the multivariate propagation of error formula as the risk is estimated with three independent distributions^14^. The uncertainties should approximately add up on the variance scale in the triple log-normal model:

$$\begin{array}{c}Var({p}_{U})\approx {(\frac{\partial {p}_{u}}{\partial {\mu }_{x}^{L}})}^{2}{\sigma }_{{\mu }_{x}^{L}}^{2}+{(\frac{\partial {p}_{u}}{\partial {\mu }_{y}^{L}})}^{2}{\sigma }_{{\mu }_{y}^{L}}^{2}+{(\frac{\partial {p}_{u}}{\partial {\mu }_{z}^{L}})}^{2}{\sigma }_{{\mu }_{z}^{L}}^{2}+{(\frac{\partial {p}_{u}}{\partial {({\sigma }_{x}^{L})}^{2}})}^{2}{\sigma }_{{({\sigma }_{x}^{L})}^{2}}^{2}\\ \,\,\,\,\,+{(\frac{\partial {p}_{u}}{\partial {({\sigma }_{y}^{L})}^{2}})}^{2}{\sigma }_{{({\sigma }_{y}^{L})}^{2}}^{2}+{(\frac{\partial {p}_{u}}{\partial {({\sigma }_{z}^{L})}^{2}})}^{2}{\sigma }_{{({\sigma }_{z}^{L})}^{2}}^{2}\end{array}$$

The derivative calculation is detailed in Appendix 2, and the variance of the risk was found to be:

$$Var({p}_{U})\approx \sum _{i=(X,Y,Z)}\,{K}_{1}\frac{{\sigma }_{i}^{2}}{{n}_{i}}+\sum _{i=(X,Y,Z)}\,{K}_{2}\frac{2{\sigma }_{i}^{4}}{({n}_{i}-1)}$$

where *n*_*i*_ is the number of data points for each input data, and

$${K}_{1}={[\phi (\frac{{\mu }_{z}^{L}-{\mu }_{x}^{L}-{\mu }_{y}^{L}}{\sqrt{{({\sigma }_{x}^{L})}^{2}+{({\sigma }_{y}^{L})}^{2}+{({\sigma }_{z}^{L})}^{2}}})\cdot \frac{1}{\sqrt{{({\sigma }_{x}^{L})}^{2}+{({\sigma }_{y}^{L})}^{2}+{({\sigma }_{z}^{L})}^{2}}}]}^{2}$$

and

$${K}_{2}={[\phi (\frac{{\mu }_{z}^{L}-{\mu }_{x}^{L}-{\mu }_{y}^{L}}{\sqrt{{({\sigma }_{x}^{L})}^{2}+{({\sigma }_{y}^{L})}^{2}+{({\sigma }_{z}^{L})}^{2}}})\cdot \frac{{\mu }_{z}^{L}-{\mu }_{x}^{L}-{\mu }_{y}^{L}}{2{(\sqrt{{({\sigma }_{x}^{L})}^{2}+{({\sigma }_{y}^{L})}^{2}+{({\sigma }_{z}^{L})}^{2}})}^{3}}]}^{2}$$

Thus, the uncertainty around the risk can be calculated mathematically in the triple log-normal case, for each source of uncertainty separately and the three combined. So, it can be actually checked that the uncertainty from the three sources sum correctly.

#### Sampling scheme: uncertainty comparison of the input variables

**Figure 2:**
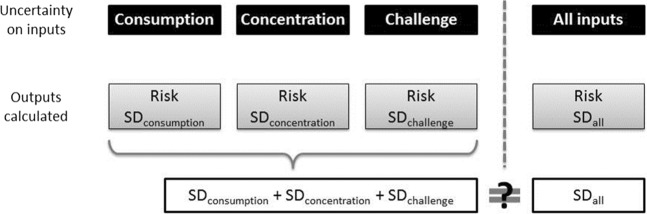
Uncertainty on the parameters sampling scheme (SD = standard deviation).

Second order Monte Carlo simulations are used. The K different sets of parameters (replications) are sampled for the three distributions with parameters calculated from the study case distributions. n simulations are performed with the K different set of parameters. In order to assess the magnitude of the uncertainty from the three distributions individually, the uncertainty around the distribution of the input parameters is integrated for one input distribution at a time while the parameters from the other inputs are fixed. Then, the risk is calculated given the uncertainty from one input distribution at a time. As the inputs are assumed to be independent, the sum of the three uncertainties is compared to the uncertainty around the risk calculated with the uncertainty associated with the three input distributions at the same time. Thus, the sum of uncertainties can be compared to the uncertainty when the uncertainties around all input parameters distributions are integrated at the same time. The parameters sampling scheme is shown on Fig. 2.

### Simulations and software

Once the sampling distribution for each parameter had been selected, the risk was calculated for each set of parameters. The number of replications (K) to evaluate the uncertainty around the risk was set to 1 000 and the number of iterations (n) per replication was set to 10 000. The simulations were performed with R software version 3.2.2^15^. The threshold distribution was fit to a survival model using the survival package (version 2.38.3) for the frequentist method and the JAGS software version 4.0.0^16^ for the Bayesian method.

### Data description

#### Concentration of unintended allergen distribution (Y)

In order to be able to compare the cases (A through D) for the risk modelling, peanut concentration data in cereal and nutrition bars were collected from peer-reviewed literature^8^. In total, the 24 data points collected were used as an input to the risk assessment.

#### Consumption (X)

The combined consumption of cereal bars in Netherlands, France and Denmark was used as an input distribution to the risk assessment^17,18^. As allergic reactions result from acute consumption on a single eating occasion, a conservative approach, i.e. the eating occasion with maximum consumption, was used.

#### Thresholds (Z)

A selection of the data described previously^19^ was used to describe the response to double blind placebo controlled food challenge (DBPCFC) with peanut protein. Thus, 158 NOAEL (No Observable Adverse Effect Level) and LOAEL (Lowest Observable Adverse Effect Level) values collected in publications^20–31^ were fitted to survival regression with Weibull or log-normal link function.

## Results

### Input distributions

#### Concentration of unintended allergen distribution (Y)

**Figure 3:**
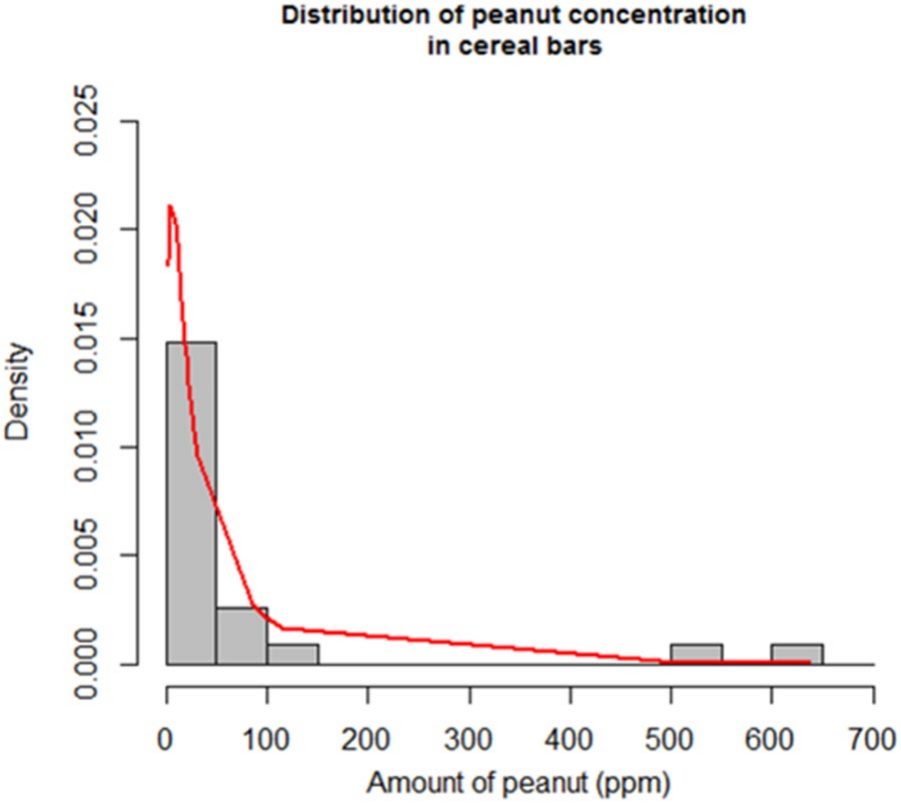
Distribution of peanut concentration in cereals bars (histogram and fitted log-normal distribution).

The mean and the standard deviation of the contamination distribution were 77 and 161 ppm respectively. Figure 3 shows the distribution of peanut concentration in cereal bars with the histogram and the log-normal distribution with mean and variance calculated from the concentration data.

Table 2 shows no difference in the μ_Y_ and σ_Y_ parameters for the log normal distribution for cases A (Spansbjerg) and B (triple log-normal). μ_Y_ is around 3.5 in both cases and σ_Y_ around 1.3. Table 2 shows that the distribution of the λ_Y_ parameter of the exponential distribution is similar when Monte Carlo simulations and Bayesian inferences are performed; the average λ_Y_ is 0.013 in cases C and D.

**Table 2:** Concentration parameters distribution for the cases A, B, C and D.

Distribution	Parameter	Mean	SD	P2.5%	Median	P97.5%
Log normal	μ_Y_ – case A	3.504	Not calculated	Not calculated	Not calculated	Not calculated
Log normal	σ_Y_ – case A	1.296	Not calculated	Not calculated	Not calculated	Not calculated
Log normal	μ_Y_ – case B	3.496	0.273	2.942	3.492	4.042
Log normal	σ_Y_ – case B	1.283	0.205	0.904	1.275	1.705
Exponential	λ_Y_ – case C	0.013	0.001	0.010	0.013	0.016
Exponential	λ_Y_ – case D	0.013	0.003	0.008	0.013	0.019

#### Consumption distribution (X)

**Figure 4:**
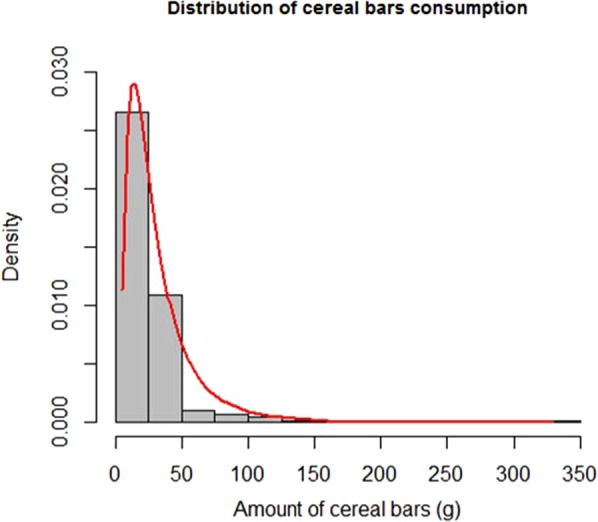
Distribution of cereal bars consumption (histogram and fitted log-normal distribution).

With the 350 participants in the three combined Food Consumption surveys consuming cereal bars, the mean and the standard deviation of cereal bar consumption were 32 g and 28 g respectively. Furthermore, 95% of the consumption lies between 1.9 g and 291.0 g. Figure 4 shows the distribution of cereal bar consumption with the histogram and the log-normal distribution with mean and variance calculated from the consumption data.

Summary statistics of the parameters of the log-normal distribution (μ_X_ and σ_X_) are presented in Table 3. In cases A (Spansbjerg), B (triple log-normal) and C μ_x_ is around −3.7 and σ_X_ is around 0.7. In case D (Rimbaud), the empirical distribution is sampled, so no distribution parameters are calculated.

**Table 3:** Consumption parameters distribution for the cases A, B and C.

Distribution	Parameter	Mean	SD	P2.5%	Median	P97.5%
Log normal	μ_x_ – case A	−3.718	Not calculated	Not calculated	Not calculated	Not calculated
Log normal	σ_X_ – case A	0.745	Not calculated	Not calculated	Not calculated	Not calculated
Log normal	μ_X_ – case B & C	−3.719	0.039	−3.796	−3.719	−3.642
Log normal	σ_X_ – case B & C	0.747	0.028	0.691	0.746	0.801

#### Threshold distribution (Z)

The summary statistics of the parameters of the Log-Normal (cases A (Spansbjerg) and B(triple log-normal)) and Weibull (cases C and D (Rimbaud)) distribution parameters fitted with a survival model are presented in Table 4.

**Table 4:** Threshold parameters distribution for the cases A, B, C and D.

Distribution	Parameter	Mean	SD	P2.5%	Median	P97.5%
Log normal	μ_Y_ – case A	4.092	Not calculated	Not calculated	Not calculated	Not calculated
Log normal	σ_Y_ – case A	2.982	Not calculated	Not calculated	Not calculated	Not calculated
Log normal	μ_Z_ – case B	4.088	0.238	3.612	4.088	4.55
Log normal	σ_Z_ – case B	2.987	0.171	2.668	2.984	3.324
Weibull	a_Z_ – case C	0.382	0.027	0.331	0.381	0.438
Weibull	b_Z_ – case C	229.621	55.099	142.773	223.298	351.067
Weibull	a_Z_ – case D	0.379	0.027	0.328	0.379	0.433
Weibull	b_Z_ – case D	225.075	51.906	139.852	219.042	341.439

In cases A (Spansbjerg) and B(triple log-normal), the estimated parameters for the log-normal distribution are similar with the average estimated coefficient for μ_Z_ is 4.09 and σ_Z_ is 2.98 in both cases. The distribution of the parameters a_Z_ and b_Z_ is similar between cases C and D (Rimbaud). The mean of distribution of a_Z_ is 0.38 in cases C and D (Rimbaud) while the mean of distribution of b_Z_ is 229.6 in case C and 225.1 in case D (Rimbaud).

### Comparison of the calculated and simulated risk of reaction to peanut in cereal bars per eating occasion

In the peanut-allergic population consuming cereal bars, the probability of allergic reaction per eating occasion after consuming a contaminated cereal bar ranges from 9.79% to 14.69% in mean, depending on the way the risk of allergic reaction is estimated. When three log-normal distributions are used (cases A (Spansbjerg) and B (triple log-normal)), the average risk of allergic reaction is similar: around 9.8–9.9%. In case B (triple log-normal), the underlying distribution of the risk can be evaluated either with mathematical calculation or with simulations. In both estimations, the risks distributions are very similar as it can be noticed in Table 5. The average probability of allergic reaction in case B (triple log-normal) was actually calculated as 9.90% (CI 95: 6.07–14.74%) and simulated as 9.90% (CI 95: 5.99–14.71%), which, considering the simulation error, is identical. In cases C and D (Rimbaud), distributions other than log-normal were used to estimate the risk of allergic reaction and it can be seen that the average risk of allergic reaction is different (higher) than in cases A and B. However, the difference in risk estimates between cases C and D (Rimbaud) and cases A (Spansbjerg) and B (triple log-normal) was expected due to the due to the characteristics of the distributions used for the risk assessments. More importantly, the risk estimates in cases C and D (Rimbaud) are similar to each other when using the same distributions for the input parameters but different simulation methods (i.e. second order Monte Carlo simulations combined with frequentist inference in case C and with Bayesian inference in case D (Rimbaud)). The average risk estimate in both cases is around 14%: in case C, the risk is on average 13.76% (CI 95: 9.94–17.97%) while in case D (Rimbaud), the risk is on average 14.69% (CI 95: 10.48–19.41%). Different sampling methods (log-normal vs bootstrap) can explain the small difference in risk.

**Table 5:** Risk distribution estimation for the 4 cases.

Case	Mean	SD	2.5%	Median	97.5%
Case A	9.79%	Not calculated	Not calculated	Not calculated	Not calculated
Case B – calculated	9.90%	2.20%	6.07%	9.75%	14.74%
Case B – simulated	9.90%	2.23%	5.99%	9.74%	14.71%
Case C – simulated	13.76%	2.09%	9.94%	13.65%	17.97%
Case D – simulated	14.69%	2.31%	10.48%	14.66%	19.41%

### Uncertainty propagation

Table 6 presents the uncertainty for cases B, C and D based on the input parameters when they are added individually or evaluated globally. In case B (triple log-normal), the individual uncertainties around the input parameters add up to similar values whether evaluated by calculation or simulation. Moreover, both the calculated global uncertainty estimates are similar to the uncertainty estimated by summing the individual uncertainties whether evaluated by calculation or simulation. Thus, it can be seen that the assumption that the three input distributions contribute independently to the uncertainty around the risk is correct. Furthermore, the rank order of the individual uncertainty inputs contributing to the uncertainty is of the same order in all cases for this example: the threshold distribution adds the most uncertainty to the risk, followed by the contamination distribution and then the consumption distribution. This observation is true whether log-normal (A, B) or Weibull (C, D) are used to fit the threshold data. The Weibull distribution contributes more uncertainty to the risk estimate than the log-normal distribution.

**Table 6:** Risk variance **(**in 10^−02^%**)** calculated by summing the uncertainties around individual input parameters compared to global uncertainty across all parameters.

Uncertainty on parameters	Case B		Case C	Case D
	Calculation	Simulation		
Consumption (X)	0.03	0.15	0.17	0.11
Contamination (Y)	2.23	2.29	0.35	1.14
Threshold (Z)	2.85	2.95	4.04	4.07
Sum of uncertainty from individual parameters	5.11	5.39	4.57	5.32
All parameters	5.28	5.37	4.36	5.37

In cases C and D, the uncertainty from the consumption distribution is similar when using a log-normal distribution or when bootstrapping in the empirical distribution. The uncertainty coming from the contamination distribution is different when using different sampling distributions for the parameter of the exponential distribution. Finally, the uncertainty from the threshold distribution is similar when using frequentist or Bayesian inference. The uncertainties are also additive in cases C and D. Furthermore, the rank order of the uncertainty contribution to the risk by each input is the same whether using a frequentist or a Bayesian approach.

Additional examples of cases B and C, using a large number of priority allergens and data from Taylor *et al*.^19^ can be found in Appendix 3. The data in Appendix 3 illustrate that the rank order of the contribution to overall uncertainty can vary depending on the allergen and total amount of data available on each of the inputs. As one would expect, there is less uncertainty from the threshold distribution when more data are available (full peanut/milk datasets) and there is more uncertainty from the threshold distribution in others with less data available (shrimp/sesame). Additionally, differences in the log-normal and Weibull threshold distribution estimates lead to more uncertainty being introduced from the use of the Weibull distribution for the threshold estimation (Appendix 3).

## Conclusion/Discussion

In this paper, four different cases were compared to estimate the risk of allergic reaction for a single eating occasion following consumption of cereal bars containing unintended peanut and to understand the mechanism of uncertainty propagation from the input parameters (amount of product consumed, concentration of peanut in product, peanut thresholds) to the risk. Bayesian and frequentist cases used to estimate parameter distributions lead to similar estimates of risk and its associated uncertainty. Using a simplified case based on three log-normal distributions, simulations and analytical calculations were compared in order to demonstrate the additive nature of all sources of uncertainty. While the Bayesian and frequentist cases produced similar results, the better familiarity of food scientists with frequentist models model suggests preferential use of the frequentist simulation technique. Moreover, the food scientists might not be familiar to the prior distribution concept which might lead to a misuse of a Bayesian implementation if the food scientists are not trained.

In the triple-log-normal case, the risk can be easily expressed mathematically as a normal distribution with the three input distribution parameters. While the triple-log-normal was used as an example, in real-life it is recommended to test several distributions (log normal, log logistic, Weibull, exponential, gamma, normal, etc) and to select the ones best fitting the data. This applies to all input distributions (threshold, consumption, contamination, etc). The distributions were selected for illustrative purposes and the comparisons could have been done with other distribution models. Furthermore, analytical calculation cannot be done in all situations as the mathematical expression of the risk gets more complex by using other distributions or when more variables are included, for example the proportion of products that are contaminated or the prevalence of allergy. Thus, when the risk expression becomes more complex, the risk can be estimated with simulations.

Choices as to the number of iterations and replications were made for the risk assessment framework of this study (10 000 iterations and 1 000 replications). However, further possibilities were explored and risks were estimated for different number of iterations (from 1 000 to 1 000 000) and replications (25, 100, 1 000 and 10 000). The variances of the risk estimates were compared for the different situations, the computation error was assessed and the computation time was also calculated. Thus, based on this investigation, the number of iterations (10 000) and replications (1 000) were found to be the best compromise between the computation time and accuracy (data not shown).

Population-based consumption data and individual threshold data are collected in separate and unrelated studies. Thus, the data collection scheme does not allow the acquisition of the matching threshold of reaction to each individual consuming cereal bars. However, it could be conceived that that the consumption and threshold of reaction are dependent of the individual. The recent MIRABEL study^32^ measured threshold of reaction during an oral food challenge for allergic individuals, as well as their corresponding frequency of consumption and the amount of certain food products to determine if doses encountered in the community are near or above the estimated threshold doses.. Additional data of this nature are needed for further analyses to investigate the possible connection between consumption and threshold at an individual level and, if necessary, implement the results in future risk assessments.

Additionally, the amount of food allergen known to cause a reaction in an individual can change over time and can be affected by different intrinsic or extrinsic factors^33^. Further calculation was integrated in the method developed in Rimbaud *et al*.^7^ (comparable to case D) which allows the investigation of the heterogeneity of the risk of allergic reaction due to a changing threshold at individual level and could similarly be integrated into frequentist methods (cases B or C).

Furthermore, the current study focuses on simulations and sampling distributions suitable for allergen risk assessment, rather than suggesting the best distribution to be used for the input variables. As a future investigation, using one simulation method which takes into account the uncertainty around the’ parameters defining the distributions, the different distribution choices for each input (threshold, consumption, contamination, etc) could be compared to recommend the distribution that gives the estimation of the risk with the lowest uncertainty.

Sensitivity analysis had already been carried out with the model developed in Spanjersberg *et al*.^9,34^. With sensitivity analysis, the input variables that have the most influence on the model output (i.e. the risk of allergic reaction) were identified^12^. The estimation of the risk of allergic reaction increased when the threshold distribution was shifted to simulate a more potent allergen, while increasing the amount consumed did not yield the same increase in the number of predicted reactions. A sensitivity analysis concerning the concentration of allergen in the food consumed was not conducted in the current study. It is expected that the influence of the input variables on the risk will be the same for the different cases presented in this paper. Further, a sensitivity analysis could be performed with the different cases presented in this paper in order to compare how the risk is impacted by a shift of input distributions.

In conclusion we have compared the four different cases that could be used in food allergen probabilistic risk assessment and found that although different mathematical formulations have been used, the overall results obtained are very similar. In addition, we have demonstrated that all methods are able to propagate uncertainty. The sources of uncertainty can be compared for the different allergens and thus, additional data can be identified which will most efficiently contribute to a reduction of uncertainty on the risk estimation.

## Supplementary information

Appendices

## Data Availability

The data that support the findings of this study are available from the iFAAM project but restrictions apply to the availability of these data, which were used under license for the current study, and so are not publicly available. Data are however available from the authors upon reasonable request and with permission of the iFAAM project.
