# Supplementary material for: Frequentist and Bayesian approaches for food allergen risk assessment: risk outcome and uncertainty comparisons
Source: Sci Rep. 2019 Dec 3;9:18206. doi: 10.1038/s41598-019-54844-1 (PMC6890679; doi:10.1038/s41598-019-54844-1)
Supplement: Supplementary file 1 — Appendices [file 41598_2019_54844_MOESM1_ESM.docx]

Frequentist and Bayesian approaches for food allergen risk assessment: risk outcome and uncertainty comparisons

# AUTHORS

Sophie BIROT ^1^, Amélie Crépet ^2^, Benjamin C. Remington ^3^, Charlotte B. Madsen ^4^, Astrid G. Kruizinga ^3^, Joseph L. Baumert ^5^ and Per B. Brockhoff ^1^

^1^ DTU Compute, Richard Petersens Plads, DK-2800 Kgs. Lyngby, Denmark

^2^ ANSES, French Agency for Food, Environmental and Occupational Health Safety, 14 rue Pierre et Marie Curie, 94701 Maisons-Alfort, France

^3^ The Netherlands Organization for Applied Scientific Research (TNO), Zeist, The Netherlands

^4^ National Food Institute, Technical University of Denmark, Denmark

# ^5^ Food Allergy Research and Resource Program, Department of Food Science & Technology, University of Nebraska, 143 Food Industry Complex, Lincoln, Nebraska 68583-0919, United States

# **Appendix 1: risk estimation with density and cumulative density (dose response) function**

In order to be able to compare the different cases, a simulation framework was defined in section 2.3.1. This framework allowed us to discard some methodological differences between the different cases. Specifically, it was highlighted that the different cases fit the threshold data in different ways, risk estimate were simulated in case B (triple log-normal) in order to validate the assumption that this does not affect the estimation. In all cases, the risk was simulated with the density function and the dose response curve for the threshold data. The distribution of risk estimates were then presented in Table VII (mean, standard deviation and some quantiles). As expected, when the uncertainties around the three inputs were included, the distributions of risk estimates are identical when taking into account simulation error. The mean risk is 9.90% calculated with the density function and 9.91% with the dose response. It is then demonstrated that using the density function or the dose response curve to describe the same distribution does not have an impact on the risk calculation. Moreover, the standard deviations are identical when calculated with the density or cumulative density function, so using one or the other way to calculate the risk of allergic reaction would not have impacted the uncertainty analysis.

| **Risk calculation** | **Mean** | **SD** | **2.5%** | **50%** | **97.5%** |
| --- | --- | --- | --- | --- | --- |
| Density function | 9.90% | 2.23% | 5.99% | 9.74% | 14.71% |
| Dose response | 9.91% | 2.20% | 6.09% | 9.77% | 14.75% |

Table VII: Risk calculation with the density function and the dose response curve in the triple log-normal case (case B)

# **Appendix 2: partial derivative calculation within the uncertainty propagation formula**

Each derivative can be then calculated separately:

$\frac{\partial p_{u}}{\partial\mu_{x}^{L}}=\phi\left( \frac{\mu_{z}^{L}-\mu_{x}^{L}-\mu_{y}^{L}}{\sqrt{(\sigma_{x}^{L})^{2}+(\sigma_{y}^{L})^{2}+(\sigma_{z}^{L})^{2}}} \right)\cdot\frac{1}{\sqrt{(\sigma_{x}^{L})^{2}+(\sigma_{y}^{L})^{2}+(\sigma_{z}^{L})^{2}}}$

$\frac{\partial p_{u}}{\partial\mu_{y}^{L}}=\phi\left( \frac{\mu_{z}^{L}-\mu_{x}^{L}-\mu_{y}^{L}}{\sqrt{(\sigma_{x}^{L})^{2}+(\sigma_{y}^{L})^{2}+(\sigma_{z}^{L})^{2}}} \right)\cdot\frac{1}{\sqrt{(\sigma_{x}^{L})^{2}+(\sigma_{y}^{L})^{2}+(\sigma_{z}^{L})^{2}}}$

$\frac{\partial p_{u}}{\partial\mu_{z}^{L}}=-\phi\left( \frac{\mu_{z}^{L}-\mu_{x}^{L}-\mu_{y}^{L}}{\sqrt{(\sigma_{x}^{L})^{2}+(\sigma_{y}^{L})^{2}+(\sigma_{z}^{L})^{2}}} \right)\cdot\frac{1}{\sqrt{(\sigma_{x}^{L})^{2}+(\sigma_{y}^{L})^{2}+(\sigma_{z}^{L})^{2}}}$

$\frac{\partial p_{u}}{\partial(\sigma_{x}^{L})^{2}}=\phi\left( \frac{\mu_{z}^{L}-\mu_{x}^{L}-\mu_{y}^{L}}{\sqrt{(\sigma_{x}^{L})^{2}+(\sigma_{y}^{L})^{2}+(\sigma_{z}^{L})^{2}}} \right)\cdot\frac{\mu_{z}^{L}-\mu_{x}^{L}-\mu_{y}^{L}}{2\left( \sqrt{(\sigma_{x}^{L})^{2}+(\sigma_{y}^{L})^{2}+(\sigma_{z}^{L})^{2}} \right)^{3}}$

$\frac{\partial p_{u}}{\partial(\sigma_{x}^{L})^{2}}=\frac{\partial p_{u}}{\partial(\sigma_{y}^{L})^{2}}=\frac{\partial p_{u}}{\partial(\sigma_{z}^{L})^{2}}$

# Appendix 3: Input uncertainty comparison for 11 different allergen risk distributions

|  | **Cashew** | **Egg** | **Hazelnut** | **Lupin** | **Milk** | **Mustard** | **Peanut** | **Sesame** | **Shrimp** | **Wheat** | **Soy Flour** |
| --- | --- | --- | --- | --- | --- | --- | --- | --- | --- | --- | --- |
| **Consumption (X)** | 0.00 | 0.04 | 0.00 | 0.00 | 0.01 | 0.10 | 0.02 | 0.01 | 0.00 | 0.01 | 0.00 |
| **Concentration (Y)** | 0.22 | 1.98 | 0.27 | 0.07 | 0.77 | 4.94 | 1.06 | 0.58 | 0.01 | 0.55 | 0.04 |
| **Threshold (Z)** | 0.45 | 1.88 | 0.21 | 0.19 | 0.29 | 12.87 | 0.17 | 3.63 | 0.14 | 1.03 | 0.21 |
| **Sum** | 0.67 | 3.90 | 0.49 | 0.27 | 1.08 | 17.91 | 1.25 | 4.22 | 0.15 | 1.59 | 0.24 |
| **All parameters** | 0.70 | 3.90 | 0.48 | 0.30 | 1.08 | 17.65 | 1.25 | 4.30 | 0.15 | 1.68 | 0.26 |

Table VIII: risk’s variance (in 10^-02^ %) when uncertainty is added to inputs’ parameters individually and all at the same time, case B -calculated

|  | **Cashew** | **Egg** | **Hazelnut** | **Lupin** | **Milk** | **Mustard** | **Peanut** | **Sesame** | **Shrimp** | **Wheat** | **Soy Flour** |
| --- | --- | --- | --- | --- | --- | --- | --- | --- | --- | --- | --- |
| **Consumption (X)** | 0.11 | 0.90 | 0.19 | 0.05 | 0.36 | 1.17 | 0.46 | 0.29 | 0.04 | 0.22 | 0.07 |
| **Concentration (Y)** | 0.32 | 2.88 | 0.46 | 0.13 | 1.11 | 6.06 | 1.47 | 0.87 | 0.05 | 0.75 | 0.10 |
| **Threshold (Z)** | 0.57 | 2.78 | 0.39 | 0.25 | 0.64 | 14.09 | 0.59 | 3.95 | 0.18 | 1.24 | 0.28 |
| **Sum** | 1.00 | 6.56 | 1.04 | 0.43 | 2.10 | 21.32 | 2.52 | 5.10 | 0.27 | 2.22 | 0.45 |
| **All parameters** | 0.83 | 4.79 | 0.68 | 0.36 | 1.41 | 18.51 | 1.65 | 4.65 | 0.20 | 1.90 | 0.33 |

Table IX: risk’s variance (in 10^-02^ %) when uncertainty is added to inputs’ parameters individually and all at the same time, case B -simulated

|  | **Cashew** | **Egg** | **Hazelnut** | **Lupin** | **Milk** | **Mustard** | **Peanut** | **Sesame** | **Shrimp** | **Wheat** | **Soy Flour** |
| --- | --- | --- | --- | --- | --- | --- | --- | --- | --- | --- | --- |
| **Consumption (X)** | 0.13 | 0.91 | 0.38 | 0.20 | 0.66 | 1.16 | 0.66 | 0.41 | 0.06 | 0.40 | 0.29 |
| **Concentration (Y)** | 0.28 | 2.08 | 0.70 | 0.36 | 1.37 | 4.77 | 1.54 | 0.84 | 0.07 | 0.94 | 0.40 |
| **Threshold (Z)** | 1.57 | 3.10 | 1.41 | 3.07 | 1.39 | 16.67 | 0.98 | 6.70 | 0.70 | 3.19 | 2.74 |
| **Sum** | 1.98 | 6.08 | 2.48 | 3.63 | 3.42 | 22.60 | 3.18 | 7.95 | 0.84 | 4.54 | 3.43 |
| **All parameters** | 1.81 | 4.38 | 1.71 | 3.29 | 2.17 | 20.64 | 1.86 | 7.26 | 0.72 | 3.75 | 2.85 |

Table X: risk’s variance (in 10^-02^ %) when uncertainty is added to inputs’ parameters individually and all at the same time, case
